# Degrading Characterization of the Newly Isolated *Nocardioides* sp. N39 for 3-Amino-5-methyl-isoxazole and the Related Genomic Information

**DOI:** 10.3390/microorganisms10081496

**Published:** 2022-07-25

**Authors:** Lei Yan, Bin Liang, Meng-Yuan Qi, Ai-Jie Wang, Zhi-Pei Liu

**Affiliations:** 1 State Key Laboratory of Microbial Resources, Institute of Microbiology, Chinese Academy of Sciences, Beijing 100101, China; shenglong00@126.com; 2 University of Chinese Academy of Sciences, Beijing 100049, China; 3 State Key Laboratory of Urban Water Resource and Environment, Shenzhen Key Laboratory of Organic Pollution Prevention and Control, School of Civil & Environmental Engineering, Harbin Institute of Technology Shenzhen, Shenzhen 518055, China; liangbin1214@hit.edu.cn (B.L.); waj0578@hit.edu.cn (A.-J.W.); 4 State Key Laboratory of Urban Water Resource and Environment, School of Environment, Harbin Institute of Technology, Harbin 150090, China; qmyqmy2008@163.com

**Keywords:** sulfamethoxazole, 3-amino-5-methyl-isoxazole, microbial degradation, *Nocardioides* sp. N39, degradation characterization

## Abstract

3-amino-5-methyl-isoxazole (3A5MI) is a persistent and harmful intermediate in the degradation of antibiotic sulfamethoxazole. It was accumulated in the environments day by day and has caused great environmental risks due to its refractory characteristic. Microbial degradation is economic and environmentally friendly and a promising method to eliminate this pollutant. In this study, a bacterial strain, *Nocardioides* sp. N39, was isolated. N39 can grow on 3A5MI as the sole carbon, nitrogen and energy resource. The effect of different factors on 3A5MI degradation by N39 was explored, including initial 3A5MI concentration, temperature, pH value, dissolved oxygen and additional carbon or nitrogen source. The degradation ability of N39 to various 3A5MI analogs was also explored. Nevertheless, the degrading ability of N39 for 3A5MI is not permanent, and long-term storage would lead to the loss of this ability. This may result from the mobile genetic elements in the bacterium according to the genomic comparison of N39 and its degrading ability-lost strain, N40. Despite this, N39 could support a lot of useful information about the degradation of 3A5MI and highlight the importance of studies about the environmental effects and potential degradation mechanism.

## 1. Introduction

As emerging environmental contaminants, residual antibiotics have attracted more and more attention in recent years. Due to their refractory metabolic characteristics, 50–100% of antibiotics were excreted by humans and animals with urine and feces [1,2]. As a result, antibiotics have been detected in most aquatic environments such as wastewater [3], surface water [4], groundwater [5] and even drinking water [6,7], as well as in many soil environments such as manure, soil and sediments [8,9,10,11]. Residual antibiotics can not only bring various direct damages to the plants and animals living in contaminated water or soil environment [9,12,13,14,15] but also break the original microbial community structure or even the ecosystem functions or enzymatic activity of bacteria [16,17,18]. Thus, finding an economic and environmentally friendly degradation method for these contaminates is crucial.

Sulfamethoxazole (SMX) is a synthetic antibiotic and is widely used in animals and humans to defend against microbial infections. It resides widely in environments on account of its common use in most countries—barely less than that of β-lactams [19,20]. The microbial degradation of SMX residing in the environment is environmentally friendly and has great applicable potential, especially with the bacterial resource that could mineralize or take SMX to metabolism cycles. However, the bacteria capable of subsisting on antibiotics are rare, and detailed information about the underlying metabolic pathways and catabolic enzymes are sparse. The degradation characteristics, kinetics and mechanism are also lacking [21,22].

To date, many reports have illustrated that 3-amino-5-methylisoxazole (3A5MI) is an important intermediate in the microbial degradation of SMX [23,24,25,26], but the refractory metabolic characteristics of 3A5MI make it intractable to SMX mineralization. 3A5MI is a derivative of isoxazole, which is an important nitrogenous heterocycle widely used in organic synthesis and has certain biological activities [27,28,29]. Considering that many degrading products of micro-pollutants may have more bio-toxicity than their parent forms [30,31], the complete degradation or mineralization of these degrading products is also of great significance. However, as far as we know, there is very little information about the degradation of 3A5MI, especially in microbial degradation. Its bio-toxicity and effect on the environment are still unknown.

As we all know, the microbial degradation of organic pollutants is greatly affected by many environmental factors such as the temperature, pH value, initial substrate concentration, dissolved oxygen and additional carbon or nitrogen source [32,33,34,35]. Ensuring the appropriate degradation conditions is important for the further implementation in microbial remediation. In previous studies, Qi et al. isolated a 3A5MI-degrading strain, *Norcardiodes* sp. N27, from activated sludge [36]. The genome character has also been reported [37]. However, the degradation characterizations and the effects of environmental factors on 3A5MI degradation are lacking, as is the degradation mechanism.

In this study, we isolated a bacterial strain, *Norcardiodes* sp. N39, from the activated sludge of a wastewater treatment plant. N39 can grow on 3A5MI as the sole carbon, nitrogen and energy resource, exhibiting formidable 3A5MI degradation potential. The effects of factors such as the initial 3A5MI concentration, pH value, temperature, dissolved oxygen and additional carbon or nitrogen source on the degradation of 3A5MI by N39 were characterized, as well as the applicability of N39 to the degradation of 3A5MI structural analogs, such as 3-amino-isoxazole (3AI), 5-methylisoxazole (5MI), isoxazole and hymexazol. The genomic information of N39 and the functional genes encoding the degradation of 3A5MI were also preliminarily explored.

## 2. Materials and Methods

### 2.1. Chemicals and Culture Medium

3A5MI (99%, Energy chemical, Shanghai, China) and 3AI (98%, Meryer, Shanghai, China) were used as the sole carbon and nitrogen source in the degradation experiments. 5MI (98%, Yuanye, Shanghai, China), isoxazole (98%, Meryer, Shanghai, China) and hymexazol (99%, Konoscience, Beijing, China) were used as the sole carbon source. The minimal salt medium (MSM) was consistent with our previous study (Yan et al., 2022). In the degradation experiments of 5MI, isoxazole and hymexazol, 10 mg/L of (NH_4_)_2_SO_4_ was supplemented in the MSM.

### 2.2. Isolation and Identification of N39 and N40

The strain N39 was isolated from the microbiomes A, which was described in detail previously [36]. LB agar plates were used for bacterial isolation. N40 was isolated from an N39 glycerol storage tube after storage at −80 °C for 6 months; it could not grow on 3A5MI as the sole carbon and nitrogen source. The 16S rRNA gene of both strains was amplified using the universal primers 27F and 1492R and sequenced in Ruibiotech (Beijing, China). The subsequent sequence alignment was performed by the Basic Local Alignment Search Tool (BLAST) and EzBioCloud online.

### 2.3. Analytic Methods

The OD_600_ was evaluated for bacterial growth, as described previously [38]. The concentration of 3A5MI was detected by a high-performance liquid chromatography system (HPLC, Agilent 1260II, USA) equipped with an Extend-C18 chromatography column (4.6 × 250 mm, 5 μm, Agilent). The mobile phase comprised 30% acetonitrile and 70% ultrapure water (0.1% formic acid, *v*/*v*) in the isocratic mode. The flow speed was set at 0.5 mL/min, the injection volume was 10 μL, the detect wavelength was 230 nm, the elution time was 10 min and the column temperature was room temperature. The detection conditions of 3AI, 5MI, isoxazole and hymexazol were the same as those of 3A5MI. All samples must have been filtered with a 0.22 μm pore size filter membrane before sample injection.

### 2.4. Degradation Experiments in Different Conditions

A single colony of N39 formed on an LB plate was inoculated into a test tube containing 5 mL of MSM medium—with 100 mg/L 3A5MI as the sole carbon and nitrogen source—and cultured at 30 °C and 160 rpm. The expansion of the culture was carried out in a 250 mL flask with 100 mL MSM with 500 mg/L 3A5MI. Cells of N39 in the stationary phase were harvested and centrifuged at 7000× *g* for 10 min, washed three times with sterile MSM and re-suspended in a sterile saline solution to a final OD_600_ of 0.2. Then, 1% (*V*/*V*) of the cell suspension was inoculated to a new 250 mL flask with 100 mL MSM for the degradation experiment. The inspection factors with an effect on 3A5MI degradation include the initial 3A5MI concentration, pH value, temperature, additional carbon or nitrogen source and dissolved oxygen. The initial 3A5MI concentration, pH value, temperature and shaking speed were set as 50 mg/L, 7.0, 30 °C and 160 rpm, respectively, when they were not the investigated factors.

The manipulation for the degradation experiments of 3AI, 5MI, isoxazole and hymexazol by N39 was the same as that for 3A5MI.

To evaluate the degradation of 3A5MI by N39 under anaerobic conditions, the experiments were carried out in an airtight bottle and filled with pure nitrogen. Inoculation and sampling were executed by a sterile injector.

### 2.5. Genomic DNA Extraction, Sequencing and Comparison

The genomic DNA of the strains N39 and N40 was extracted using a bacterial DNA kit (Omega Bio-Tek, Norcross, GA, USA), according to the instructions of the manufacturer. The SQK-LSK109 kit was used for library preparation following the 1D genomic DNA by the ligation protocol [39]. The genomic DNA was sequenced by a combination of next-generation sequencing by an Illumina platform and third-generation sequencing by a Nanopore sequencing platform. Filtered reads were assembled using the software Unicycler (0.4.8). Coding genes were predicted by the software Prokka (1.1.2) and annotated by the UniProt, KEGG, GO, RefSeq, Pfam, COG and TIGERfam databases. The comparison of the genomic DNA was accomplished by Rapid Annotation using Subsystem Technology (RAST) online. The single nucleotide polymorphism (SNP) and indel between the strains N39 and N40 were analyzed by the software MUMmer (version 4.x) [40]. The whole genomic sequencing was completed by Benagen (Wuhan, China).

## 3. Results and Discussion

### 3.1. Isolation of Strains N39 and N40

Besides the above-mentioned bacterial strain (N27) isolated in the microbiomes [37], another strain capable of growing on 3A5MI as the sole carbon and nitrogen source was also isolated and named *Norcardiodes* sp. N39. The morphological characterization and 16S rRNA sequence of N39 were the same as those of the strain N27 [38], but the genomic information was not identical (Table 1). After storing N39 in glycerol tubes for 6 months at −80 °C, a new strain with no ability to grow on 3A5MI as the sole carbon and nitrogen source was obtained and termed *Norcardiodes* sp. N40. The strains N39 and N40 shared identical 16S rRNA sequences and morphological characteristics but exhibited completely different abilities in terms of 3A5MI degradation.

### 3.2. Effect of Different Factors on 3A5MI Degradation by N39

#### 3.2.1. Effect of the Initial 3A5MI Concentration on the Degradation Ability of N39

**Figure 1:**
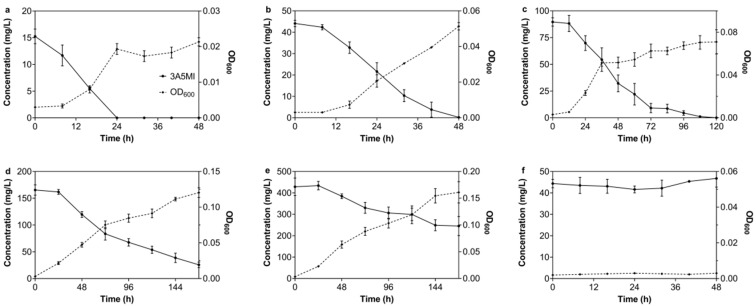
Effect of initial 3A5MI concentrations on its degradation by *Nocardioides* sp. N39. (**a**) 15 mg/L; (**b**) 45 mg/L; (**c**) 90 mg/L; (**d**) 165 mg/L; (**e**) 430 mg/L; (**f**) control checks of 3A5MI degradation without the inoculation of N39 and the growth of N39 without the addition of 3A5MI and any other carbon source. Symbols are shown in the figure.

The degradation of 3A5MI by N39 was tested at a concentration ranging from 15.26 ± 1.36 to 428.31 ± 40.80 mg/L. As shown in Figure 1, the great 3A5MI-degrading ability of N39 was exhibited in both lower and higher initial 3A5MI contents. The initial concentration of 15 mg/L 3A5MI was completely degraded within 24 h, accompanied by N39 grown from OD_600_ = 0.003 to 0.022. The biomass of N39 also reached its peak when 3A5MI was completely degraded (Figure 1a). Their synchronization directly indicated that 3A5MI was used by N39 as a substrate source for growth. When the initial 3A5MI concentration was 165.33 mg/L, 84.51 ± 3.10% of 3A5MI was catabolized in 168 h, and the OD_600_ increased from 0.003 ± 0.000 to 0.120 ± 0.007 (Figure 1d), indicating a slower catabolic rate compared to the lower initial concentration. A similar result was obtained when the initial concentration was 428.31 mg/L; about 42.80% of 3A5MI was degraded within 168 h (Figure 1e). Interestingly, when the initial concentration was higher than 50 mg/L, the growth of N39 came to a standstill in the first 8 h (Figure 1b–e). This may be ascribed to the amount of introduced N39 not being sufficient to cause immediate and significant degradation for high concentrations of 3A5MI.

Moreover, the degradation of 3A5MI in the control (without the inoculation of N39; represented as a solid line with filled circles in Figure 1f) was also determined. The result indicated that there was no abiotic degradation of 3A5MI. Likewise, no growth of N39 was observed in another control without the addition of 3A5MI or any other carbon source (represented as a dotted line with black rhombuses in Figure 1f), supporting the credibility of our experimental results.

#### 3.2.2. Effect of Temperature on the Degradation of 3A5MI by N39

The degradation of 3A5MI by N39 was conducted at 20 °C, 25 °C, 30 °C, 35 °C and 40 °C, respectively. The results indicated a high degradation efficiency from 30 °C to 35 °C (Table 2). Approximately 50 mg/L of 3A5MI was almost totally catabolized within 48 h during this temperature range. However, the growth of N39 was displayed in different patterns. The growth yields of N39 at 30 and 35 °C in 48 h were 11.03 × 10^−4^ ± 0.90 × 10^−4^ and 8.86 × 10^−4^ ± 0.71 × 10^−4^ AU/mg, obviously larger than the 3.43 × 10^−4^ ± 8.39 × 10^−4^, 4.28 × 10^−4^ ± 0.06 × 10^−4^ and 0.00 ± 0.00 AU/mg observed under 20, 25 and 40 °C, respectively. The growth rate exhibited the same trend. The growth rates of N39 at 30 and 35 °C in 48h were 10.07 × 10^−4^ ± 0.43 × 10^−4^ and 7.92 × 10^−4^ ± 0.42 × 10^−4^ AU/h, higher than those observed at 20, 25 and 40 °C: 2.43 × 10^−4^ ± 0.48 × 10^−4^, 3.75 × 10^−4^ ± 0.55 × 10^−4^ and 0.00 ± 0.00 AU/h, respectively. The catabolic ability of N39 for 3A5MI under 20 °C performed sluggishly. It was obviously strengthened after being cultured for 32 h. The degradation speed of 0.86 mg/L/h during 32–48 h was obviously higher than the 0.16 mg/L/h observed during 0–32 h. The temperature of 40 °C was not appropriate for growing N39. No increase in the OD_600_ values was observed during the cultivation, nor a decrease in the 3A5MI concentration. This is inconsistent with the previous report that the *Nocardioides* sp. strain JQ2195 was functional for cotinine degrading at 40 °C [41], and it is similar to the result that the degradation of atrazine by the *Nocardioides* sp. strain EAA-3 was negligible at 45 °C [42]. These results revealed that the appropriate growth conditions of the strains in *Nocardioides* sp. were diverse.

Briefly, 30–35 °C is the appropriate temperature range for both 3A5MI degradation and N39 growth, and 30 °C is the most applicable. Low temperatures would decline the efficiency, and high temperatures are not suitable for the subsistence of N39.

#### 3.2.3. Effect of the pH Value on the Degradation of 3A5MI by N39

The growth yield and growth rate of N39 with 3A5MI as the sole carbon and nitrogen source were explored under different pH values from 5.0 to 9.0. As shown in Table 3, N39 exhibited various growth patterns under different pH values. Obviously, the nature or alkaline solution is a preferential condition for N39 growth and 3A5MI degradation compared to the acid condition. The initial 44.53 ± 1.95 mg/L 3A5MI was totally catabolized within 32 h at pH 9.0, while only 19.05 ± 3.61% of 3A5MI was degraded in 48 h at pH 5.0. The growth of N39 under acidic conditions also obviously fell behind that under alkaline and natural conditions. The growth yields of N39 were larger than 9.5 × 10^−4^ AU/mg in 48 h under the nature and alkaline conditions, while they were less than 7.5 × 10^−4^ AU/mg at the same time under the acid condition. The changes in the growth rate were consistent with the growth yields. The *Nocardioides* sp. N39 grows well in the nature and alkaline conditions but not in the acid condition. Especially, the growth rate in 48 h was 1.04 × 10^−4^ ± 0.42 × 10^−4^ AU/h when the pH was 5.0, obviously less than that under higher pH conditions.

Noticeably, OD_600_ increased most significantly at pH 7.0, implying that it is the optimum pH value for the growth of N39, in accordance with the description of *Nocardioides seonyuensis* sp. nov., *Nocardioides euryhalodurans* sp. nov. and *Nocardioides eburneiflavus* sp. nov. [43]. However, 3A5MI degradation is more efficient under alkaline conditions, as distinguished from the optimal conditions for N39 growth.

#### 3.2.4. Effect of Dissolved Oxygen on the Degrading Ability of N39

**Figure 2:**
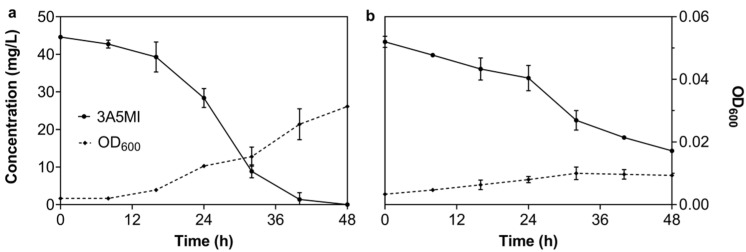
Degradation of 3A5MI by the *Nocardioides* sp. N39 under stationary culture (**a**) and anaerobic culture (**b**). Symbols are shown in the figure.

To verify the oxygen demand of N39 during the degradation of 3A5MI, the degradation experiments under anaerobic (closed bottles filled with pure nitrogen) and mionectic conditions (air-permeable but statically cultured) were executed. The results in Figure 2 illustrate that N39 was functional under both mionectic and anaerobic conditions. About 45 mg/L of 3A5MI was degraded within 48 h under mionectic conditions, and the growth of N39 increased from OD_600_ 0.002 to 0.031 (Figure 2a). Obvious degradation was also observed under the anaerobic conditions; 63.24 ± 9.61% of 3A5MI was catabolized within 48 h (Figure 2b). N39 grew slightly as the OD_600_ increased from 0.002 to 0.008 ± 0.002. These results suggested that N39 was functional in both oxygen-enriched and hypoxic conditions, showing its strong potential for environmental remediation, especially in deep water or soil.

Furthermore, we found that there are 10 anaerobic respiratory reductases in the annotated genome of strain N39 that take a part of terminal electron acceptors in anaerobic respiration. They consisted of two ferredoxin reductases, five flavodoxin reductases and vanillate O-demethylase oxidoreductase, supporting that the theoretical foundation for N39 was functional in degrading 3A5MI under anaerobic conditions.

#### 3.2.5. Effect of Additional Carbon or Nitrogen on the Degrading Ability of N39

**Figure 3:**
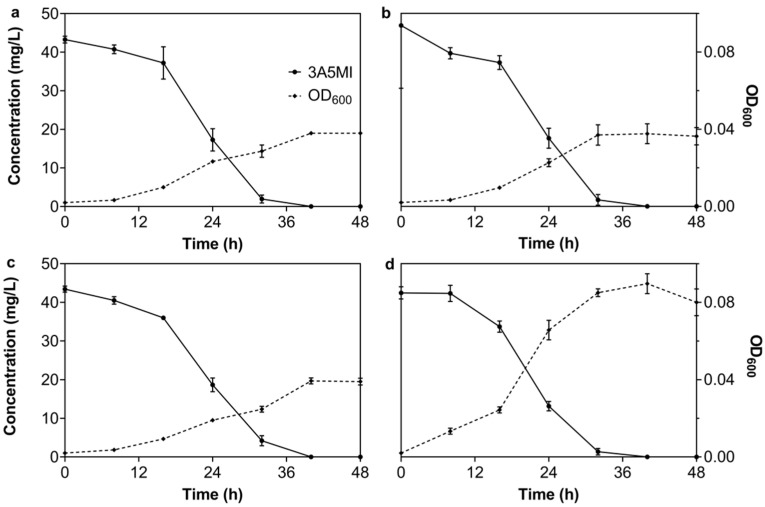
Effect of an additional carbon or nitrogen source on 3A5MI degradation by *Nocardioides* sp. N39. (**a**) 50 mg/L sodium pyruvate; (**b**) 50 mg/L ammonium sulfate; (**c**) 50 mg/L sodium pyruvate and 50 mg/L ammonium sulfate; (**d**) 50 mg/L yeast extraction. Symbols are shown in the figure.

It was reported that an additional carbon source could accelerate the degradation of antibiotics in the environment [44,45]. To ensure the effect of an additional carbon or nitrogen source on the degradation of 3A5MI by N39, degradation experiments were conducted with the addition of 50 mg/L sodium pyruvate, 50 mg/L ammonium sulfate and 50 mg/L yeast extraction, respectively, as well as 50 mg/L sodium pyruvate and 50 mg/L ammonium sulfate. It was concluded that the degradation patterns of 3A5MI were similar among the experiment groups and were not obviously affected by the addition of an individual carbon source (Figure 3a), an individual nitrogen source (Figure 3b), a mixture of carbon and nitrogen sources (Figure 3c) or a complex of carbon, nitrogen and growth factors (Figure 3d). Confusingly, the addition of pyruvate and ammonium sulfate did not enhance the growth of N39 within 48 h, although this bacterium owned all the metabolic enzymes of pyruvate (Supplementary Figures S1 and S2). Combined with the phenomenon that the N39 grown with sodium pyruvate and ammonium sulfate as the sole carbon and nitrogen source occurred in 4–5 days and that the OD_600_ was not increased in the first 2 days, this may imply that N39 utilized 3A5MI as the preferential carbon source over pyruvate. Moreover, the addition of yeast extraction obviously enhanced the growth of N39 as the OD_600_ increased from 0.020 ± 0.000 to 0.080 ± 0.009 within 48 h, but the degradation of 3A5MI was not obviously affected compared to the assays with no additional carbon or nitrogen source (Figure 1c). These results demonstrate that N39 was still functional in degrading 3A5MI rather than unitizing other carbon sources, exhibiting a great 3A5MI removal capability and potential in complex environments.

#### 3.2.6. The Degrading Spectrum of N39 for 3A5MI Analogs

**Figure 4:**
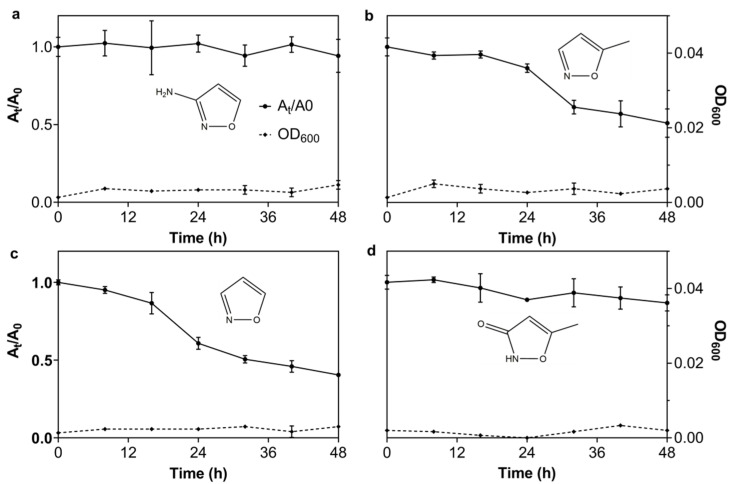
Degrading spectrum of N39 for 3A5MI analogs. (**a**) 3AI; (**b**) 5MI; (**c**) isoxazole; (**d**) hymexazol. The left ordinate represents the ratio of the peak area at the t-hour to the initial peak area (detected wavelength was 230 nm). Symbols are shown in the figure.

To investigate the applicability of N39 for degrading 3A5MI analogs, its degrading ability with respect to other 3A5MI analogs was tested, including 3AI, 5MI, isoxazole and hymexazol. The results in Figure 4 show that the concentration of 5MI and isoxazole decreased obviously within 48 h, but N39 did not grow. While the decrease in 3AI was negligible and the decrease in hymexazol was slight, N39 did not grow either. This phenomenon may be ascribed to the fact that the enzymes in N39 were functional in degrading them, but the degradation products could not be utilized as growth substrates. Alternatively, it could be ascribed to the possibility of the hysteresis of bacterial growth compared to the substrate utilization. In general, the degradation of the 3A5MI analogs implied the great potential of N39 in the bioremediation of the environments contaminated by such compounds.

In conclusion, *Nocardioides* sp. N39 exhibited a great 3A5MI degrading ability under different conditions and was also functional to some 3A5MI analogs.

### 3.3. Genomic Characteristic of the Strains N39 and N40

**Figure 5:**
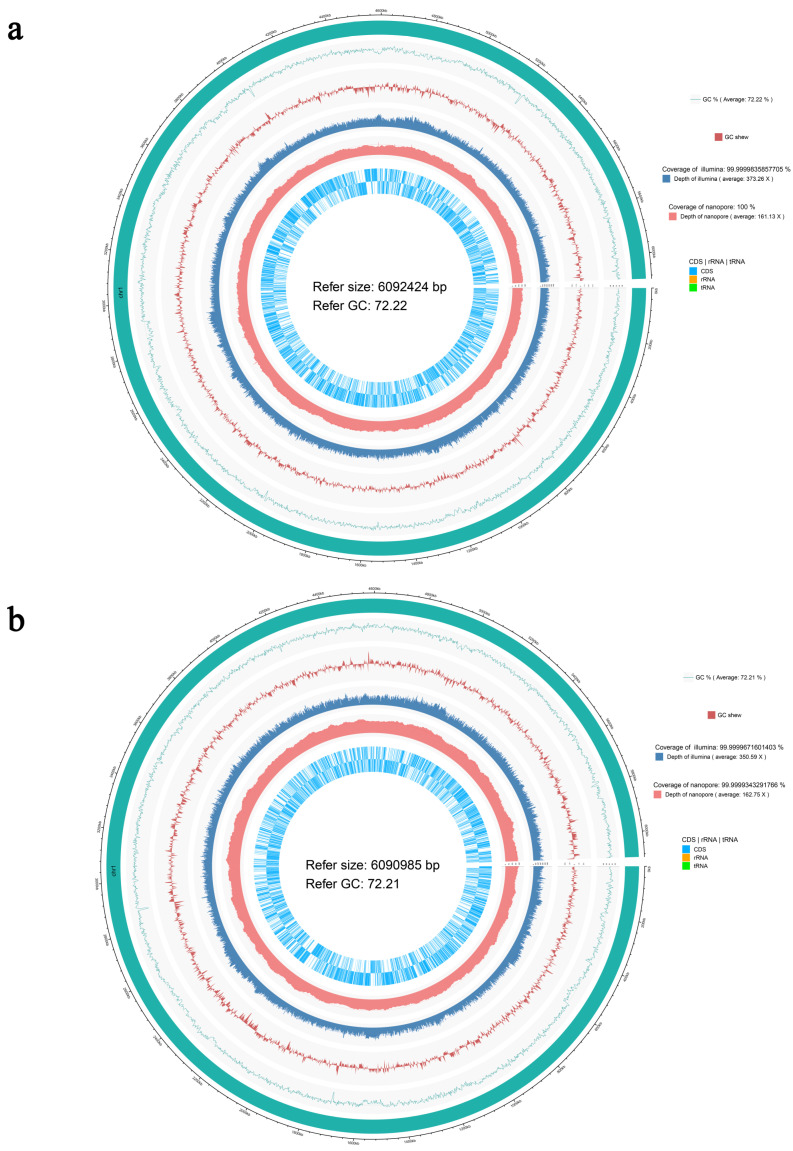
The nuclear genome circle diagrams of *Nocardioides* sp. N39 (**a**) and N40 (**b**). From outside to inside: the reference species genome sequence information; the GC content curve of the reference genome sequence; the GC skew curve of the reference genome sequence; Illumina sequencing depth and coverage information; Nanopore sequencing depth and coverage information; the gene coding region (CDS) and non-coding RNA regions (rRNA, tRNA) in the reference genome, respectively.

As described previously, the functional character of N39 grown on 3A5MI as the sole carbon and nitrogen source was not stably inherited. To explore the cause of the degradation function loss and the real functional genes of N39 for assimilating the carbon in 3A5MI, the complete genomes of N39 and N40 were sequenced and compared. The genome sequence sizes of N39 and N40 are 6,268,928 and 6,263,677 bp, with GenBank accession numbers in NCBI of CP097984-CP097987 and CP096276-CP096278, respectively. The nuclear genome circle diagram was displayed in Figure 5. Their high GC contents (both at 72.2%) were similar to the description of bacteria classified to the genus *Nocardioides* [46,47]. A total of four and three contigs were assembled in N39 and N40, respectively, and no gaps were found. There were 6157 genes, and 6076 coding sequences were predicted in N39, while 6152 genes and 6071 coding sequences were predicted in N40.

As seen in Table 1, when compared to the genome of N40, N39 contains three plasmids, including the extra one with a length of 3820 bp. However, the whole sequence in this extra plasmid would align with the chromosome of N39 and N40 with 100% identity. The plasmid II of N39 was identical to the plasmid II of N40, and the plasmid I of N39 was 99% (110937/110945), identical to the plasmid I of N40. This means that the loss of plasmids may not be the true reason for its loss in 3A5MI catabolic ability.

When compared the chromosome sequence of these two strains, it was found that 60 genes of N39 have no hits compared to the genomic sequence of N40, and 68 genes have an identity <100% (details are listed in Supplementary Table S1). Of the 60 genes presented in N39 but not in N40, 19 genes encode the mobile element protein, and 4 genes encode the integrase catalytic subunit. A previous study has shown that inserted mobile elements of various types and sizes contribute to the control of gene transcription [48]. This supportiveness led us to speculate that the genes encoding the functional enzymes that catabolize 3A5MI are damaged during transcription compared to N39; therefore, the inability to catabolize 3A5MI was observed in strain N40. The last 37 genes contained in the 60 genes in N39 with no hits to N40 were unannotated with RAST. This means that the functional enzymes may be new and have not been reported previously.

In addition, SNP/indel analysis was performed on these two bacterial genomes. The detailed results in Supplementary Table S2 indicate that there are 355 SNP loci between N39 and N40, all of which were located in their chromosomes. There were 120 SNP loci (distributed in 7 intergenic regions and in the inside of 14 different genes) and 235 indel loci found in the genome of N39. This information can provide some useful clues for finding functional genes in the degradation of 3A5MI, but further exploration and research are still required.

## 4. Conclusions

3A5MI is a persistent and harmful intermediate in the degradation and synthesis of antibiotic sulfamethoxazole. It is more and more accumulated in the environment; therefore, its elimination is of great urgency. In this study, a bacterial strain, *Nocardioides* sp. N39, was isolated. The strain N39 could grow on 3A5MI as the sole carbon, nitrogen and energy resource. The optimal conditions for the degradation of 3A5MI by N39 are an initial 3A5MI concentration below 100 mg/L, an alkaline solution, a temperature of 25–35 °C and aerobic conditions. An additional carbon or nitrogen source did not obviously affect the degradation. N39 also showed a degrading ability to some 3A5MI analogs, demonstrating its potential in the remediation of a contaminated environment. However, the function of 3A5MI degradation in N39 is not permanent, and long-term storage can easily lead to function loss. This may result from the mobile genetic elements in this bacterium according to the genomic comparison of N39 and N40, which lose the function of growing with 3A5MI as the sole carbon, nitrogen and energy source.

## Figures and Tables

**Table 1:** The genomic information of *Norcardiodes* sp. N39, N40 and N27.

Strain	Genes	CDS	Gaps	Sequence	Contig Length (bp)	GC Content (%)	Reference
N39	6157	6076	0	Chromosome	6,092,424	72.28	This study
				Plasmid I	110,939	69.60	
				Plasmid II	61,745	69.89	
				Plasmid III	3820	78.27	
N40	6152	6071	0	Chromosome	6,090,985	72.29	This study
				Plasmid 1	110,947	69.60	
				Plasmid 2	61,745	69.89	
N27	6658	6603		Chromosome	6,104,465	72.29	[37]
				Plasmid A	294,133	67.26	
				Plasmid B	109,667	69.95	
				Plasmid C	80,343	69.90	

**Table 2:** Effect of temperature on the growth of *Nocardioides* sp. N39 with 3A5MI as the sole carbon and nitrogen source.

Growth Yield (×10^−4^ AU/mg)	20 °C	25 °C	30 °C	35 °C	40 °C
0 h	0.00 ^a^ ± 0.00 ^b^	0.00 ± 0.00	0.00 ± 0.00	0.00 ± 0.00	0.00 ± 0.00
8 h	0.00 ± 0.00	0.00 ± 0.00	0.00 ± 0.00	0.00 ± 0.00	0.00 ± 0.00
16 h	2.08 ± 3.84	0.56 ± 0.96	3.67 ± 0.68	4.56 ± 2.06	0.00 ± 0.00
24 h	1.23 ± 1.30	2.55 ± 1.49	7.10 ± 0.52	6.95 ± 1.29	0.00 ± 0.00
32 h	1.51 ± 1.58	2.84 ± 0.35	8.60 ± 1.00	7.33 ± 0.39	0.00 ± 0.00
40 h	2.21 ± 4.08	2.38 ± 0.40	9.77 ± 0.91	7.77 ± 0.32	0.00 ± 0.00
48 h	3.43 ± 8.39	4.28 ± 0.06	11.03 ± 0.90	8.86 ± 0.71	0.00 ± 0.00
Growth rate (×10^−4^ AU/h)	20 °C	25 °C	30 °C	35 °C	40 °C
0 h	0.00 ± 0.00	0.00 ± 0.00	0.00 ± 0.00	0.00 ± 0.00	0.00 ± 0.00
8 h	0.00 ± 1.25	0.00 ± 0.00	0.00 ± 0.00	0.00 ± 0.00	0.00 ± 0.00
16 h	0.42 ± 0.36	0.21 ± 0.36	2.71 ± 1.30	2.50 ± 0.63	0.00 ± 0.00
24 h	0.42 ± 0.00	0.83 ± 0.42	7.36 ± 1.46	6.39 ± 0.96	0.00 ± 0.00
32 h	0.21 ± 0.18	1.88 ± 0.00	10.00 ± 2.44	7.40 ± 0.18	0.00 ± 0.00
40 h	1.83 ± 0.29	1.83 ± 0.38	9.83 ± 1.23	7.58 ± 0.14	0.00 ± 0.00
48 h	2.43 ± 0.48	3.75 ± 0.55	10.07 ± 0.43	7.92 ± 0.42	0.00 ± 0.00

Note: a ± b represents the average ± standard deviation of three replicates. The ×10^−4^ in the parentheses of the table header represent that the values of the growth yield and growth rate in the table were multiplied by 10,000.

**Table 3:** Effect of pH conditions on the growth of *Nocardioides* sp. N39 with 3A5MI as the sole carbon and nitrogen source.

Growth Yield (×10^−4^ AU/mg)	pH = 5.0	pH = 6.0	pH = 7.0	pH = 8.0	pH = 9.0
0 h	0.00 ^a^ ± 0.00 ^b^	0.00 ± 0.00	0.00 ± 0.00	0.00 ± 0.00	0.00 ± 0.00
8 h	0.00 ± 0.00	4.56 ± 5.66	0.00 ± 0.00	0.00 ± 0.00	0.00 ± 0.00
16 h	0.00 ± 0.00	0.35 ± 2.32	3.67 ± 0.68	1.91 ± 1.51	3.22 ± 0.88
24 h	0.00 ± 0.00	2.82 ± 1.11	7.10 ± 0.52	5.07 ± 1.90	6.03 ± 1.10
32 h	0.00 ± 0.00	3.76 ± 0.90	8.60 ± 1.00	7.10 ± 1.52	8.41 ± 1.35
40 h	0.68 ± 2.21	5.42 ± 1.34	9.77 ± 0.91	8.73 ± 1.05	9.07 ± 0.41
48 h	6.35 ± 4.35	7.32 ± 1.13	11.03 ± 0.90	9.55 ± 1.52	9.52 ± 0.44
Growth rate (×10^−4^ AU/h)	pH = 5.0	pH = 6.0	pH = 7.0	pH = 8.0	pH = 9.0
0 h	0.00 ± 0.00	0.00 ± 0.00	0.00 ± 0.00	0.00 ± 0.00	0.00 ± 0.00
8 h	0.00 ± 0.00	0.00 ± 0.00	0.00 ± 0.00	0.00 ± 0.00	0.00 ± 0.00
16 h	0.00 ± 0.00	0.00 ± 0.63	2.71 ± 1.30	0.83 ± 0.36	1.88 ± 0.00
24 h	0.00 ± 0.00	1.39 ± 0.64	7.36 ± 1.46	4.44 ± 0.87	7.50 ± 1.25
32 h	0.00 ± 0.00	2.81 ± 0.94	10.00 ± 2.44	8.65 ± 0.18	11.67 ± 1.57
40 h	0.17 ± 0.38	5.00 ± 1.50	9.83 ± 1.23	10.17 ± 0.52	10.08 ± 0.14
48 h	1.04 ± 0.42	6.46 ± 0.63	10.07 ± 0.43	9.24 ± 0.64	8.82 ± 0.32

Note: a ± b represents the average ± standard deviation of three replicates. The growth yield and growth rate in the table were amplified 10,000-fold.

## Supplementary Materials

The following supporting information can be downloaded at: https://www.mdpi.com/article/10.3390/microorganisms10081496/s1, Table S1. The genes in N39 distinct to N40. Table S2. The genomic SNP/indel analysis between strain N39 and N40. Figure S1. The metabolic pathway of pyruvate in N39 obtained from KEGG database. Figure S2. The metabolic pathway of citrate cycle in N39 obtained from KEGG.
