# Supplementary material for: Degrading Characterization of the Newly Isolated Nocardioides sp. N39 for 3-Amino-5-methyl-isoxazole and the Related Genomic Information
Source: Microorganisms. 2022 Jul 25;10(8):1496. doi: 10.3390/microorganisms10081496 (PMC9329766; doi:10.3390/microorganisms10081496)
Supplement: Supplementary file 1 [file microorganisms-10-01496-s001.zip › microorganisms-1781645-supplementary.pdf]

Degrading characterization of newly isolated *Nocardioides* sp. N39 for 3-amino-5-methyl-isoxazole and its genomic information

Lei Yan [1,2], Bin Liang [3], Meng-Yuan Qi [4], Ai-Jie Wang [3,4] and Zhi-Pei Liu [1]*

[1] State Key Laboratory of Microbial Resources, Institute of Microbiology, Chinese Academy of Sciences, Beijing 100101, China; shenglong00@126.com (L.Y.); liuzhp@im.ac.cn (Z.-P. L.)

[2] University of Chinese Academy of Sciences, Beijing 100049, China

[3] State Key Laboratory of Urban Water Resource and Environment, Shenzhen Key Laboratory of Organic Pollution Prevention and Control, School of Civil & Environmental Engineering, Harbin Institute of Technology Shenzhen, Shenzhen 518055, China; liangbin1214@hit.edu.cn (B. L.); waj0578@hit.edu.cn (A.-J.W.)

[4] State Key Laboratory of Urban Water Resource and Environment, School of Environment, Harbin Institute of Technology, Harbin 150090, China; 382965860@qq.com (M.-Y.Q.)

* Correspondence: liuzhp@im.ac.cn; Tel.: +86-10-64806081

Table S1. The genes in N39 distinct to N40

Length	Gene ID in N39	Annotated function	Corresponding gene ID in N40	Percent identity
262	fig|2045.14.peg.103	hypothetical protein		0
60	fig|2045.14.peg.710	hypothetical protein		0
360	fig|2045.14.peg.714	hypothetical protein		0
53	fig|2045.14.peg.859	hypothetical protein		0
118	fig|2045.14.peg.1134	hypothetical protein		0
38	fig|2045.14.peg.1169	Mobile element protein		0
349	fig|2045.14.peg.1213	hypothetical protein		0
437	fig|2045.14.peg.1214	hypothetical protein		0
456	fig|2045.14.peg.1294	hypothetical protein		0
63	fig|2045.14.peg.1491	hypothetical protein		0
41	fig|2045.14.peg.2090	hypothetical protein		0
102	fig|2045.14.peg.2096	hypothetical protein		0
159	fig|2045.14.peg.2208	hypothetical protein		0
415	fig|2045.14.peg.2485	Mobile element protein		0
415	fig|2045.14.peg.2489	Mobile element protein		0
44	fig|2045.14.peg.2528	hypothetical protein		0
101	fig|2045.14.peg.2637	hypothetical protein		0
415	fig|2045.14.peg.2712	Mobile element protein		0
415	fig|2045.14.peg.2752	Mobile element protein		0
74	fig|2045.14.peg.2765	hypothetical protein		0
415	fig|2045.14.peg.2779	Mobile element protein		0
415	fig|2045.14.peg.2782	Mobile element protein		0
415	fig|2045.14.peg.2791	Mobile element protein		0
48	fig|2045.14.peg.2797	hypothetical protein		0
58	fig|2045.14.peg.2799	hypothetical protein		0
51	fig|2045.14.peg.2805	hypothetical protein		0
78	fig|2045.14.peg.2814	hypothetical protein		0
81	fig|2045.14.peg.2815	hypothetical protein		0
71	fig|2045.14.peg.2816	hypothetical protein		0
45	fig|2045.14.peg.2817	hypothetical protein		0
91	fig|2045.14.peg.2948	hypothetical protein		0
94	fig|2045.14.peg.2949	hypothetical protein		0
133	fig|2045.14.peg.3003	hypothetical protein		0
50	fig|2045.14.peg.3421	hypothetical protein		0
415	fig|2045.14.peg.3735	Mobile element protein		0
413	fig|2045.14.peg.3745	Mobile element protein		0
207	fig|2045.14.peg.3771	hypothetical protein		0
57	fig|2045.14.peg.3772	hypothetical protein		0
52	fig|2045.14.peg.4357	hypothetical protein		0

415	fig|2045.14.peg.4506	Mobile element protein		0
413	fig|2045.14.peg.4516	Mobile element protein		0
413	fig|2045.14.peg.4532	Mobile element protein		0
290	fig|2045.14.peg.4623	integrase catalytic subunit		0
415	fig|2045.14.peg.4657	Mobile element protein		0
415	fig|2045.14.peg.4669	Mobile element protein		0
415	fig|2045.14.peg.4679	Mobile element protein		0
413	fig|2045.14.peg.4703	Mobile element protein		0
303	fig|2045.14.peg.4731	Mobile element protein		0
173	fig|2045.14.peg.4744	hypothetical protein		0
103	fig|2045.14.peg.4758	hypothetical protein		0
303	fig|2045.14.peg.4762	Mobile element protein		0
42	fig|2045.14.peg.4776	hypothetical protein		0
290	fig|2045.14.peg.4783	integrase catalytic subunit		0
348	fig|2045.14.peg.4785	hypothetical protein		0
290	fig|2045.14.peg.4794	integrase catalytic subunit		0
290	fig|2045.14.peg.4919	integrase catalytic subunit		0
48	fig|2045.14.peg.5055	hypothetical protein		0
47	fig|2045.14.peg.5348	hypothetical protein		0
77	fig|2045.14.peg.5627	hypothetical protein		0
40	fig|2045.14.peg.5831	hypothetical protein		0
372	fig|2045.14.peg.73	hypothetical protein	fig|2045.18.peg.3996	99.46
41	fig|2045.14.peg.173	hypothetical protein	fig|2045.18.peg.4096	97.5
637	fig|2045.14.peg.398	Osmosensitive K+ channel histidine kinase KdpD (EC 2.7.3.-)	fig|2045.18.peg.4322	99.74
616	fig|2045.14.peg.445	Mlr6999 protein	fig|2045.18.peg.4369	99.84
262	fig|2045.14.peg.572	hypothetical protein	fig|2045.18.peg.4497	99.61
45	fig|2045.14.peg.706	hypothetical protein	fig|2045.18.peg.4633	97.73
350	fig|2045.14.peg.708	hypothetical protein	fig|2045.18.peg.4635	99.71
621	fig|2045.14.peg.784	hypothetical protein	fig|2045.18.peg.4709	99.81
279	fig|2045.14.peg.867	hypothetical protein	fig|2045.18.peg.4791	99.64
50	fig|2045.14.peg.934	hypothetical protein	fig|2045.18.peg.4858	97.96
1013	fig|2045.14.peg.1073	hypothetical protein	fig|2045.18.peg.4998	99.9
144	fig|2045.14.peg.1192	hypothetical protein	fig|2045.18.peg.5119	99.3
225	fig|2045.14.peg.1409	hypothetical protein	fig|2045.18.peg.5336	99.49
197	fig|2045.14.peg.1651	hypothetical protein	fig|2045.18.peg.5578	99.35
384	fig|2045.14.peg.1858	hypothetical protein	fig|2045.18.peg.5785	99.74
308	fig|2045.14.peg.1889	hypothetical protein	fig|2045.18.peg.5816	99.5
252	fig|2045.14.peg.1955	hypothetical protein	fig|2045.18.peg.5882	99.6
318	fig|2045.14.peg.2040	Transcriptional regulator, LysR family	fig|2045.18.peg.5967	99.66
155	fig|2045.14.peg.2054	hypothetical protein	fig|2045.18.peg.5981	99.35

587	fig|2045.14.peg.2073	hypothetical protein	fig|2045.18.peg.6000	99.83
215	fig|2045.14.peg.2117	hypothetical protein	fig|2045.18.peg.6043	99.27
83	fig|2045.14.peg.2212	hypothetical protein	fig|2045.18.peg.6139	98.51
333	fig|2045.14.peg.2354	Glycine oxidase ThiO (EC 1.4.3.19)	fig|2045.18.peg.278	99.39
40	fig|2045.14.peg.2488	hypothetical protein	fig|2045.18.peg.412	97.44
226	fig|2045.14.peg.2492	hypothetical protein	fig|2045.18.peg.416	98.75
153	fig|2045.14.peg.2522	hypothetical protein	fig|2045.18.peg.447	98.73
782	fig|2045.14.peg.2523	ATP-dependent DNA helicase UvrD/PcrA (EC 3.6.4.12)	fig|2045.18.peg.448	99.87
333	fig|2045.14.peg.2588	hypothetical protein	fig|2045.18.peg.512	99.69
526	fig|2045.14.peg.2597	Cys-tRNA(Pro) deacylase YbaK	fig|2045.18.peg.521	99.8
205	fig|2045.14.peg.2666	hypothetical protein	fig|2045.18.peg.592	99.41
216	fig|2045.14.peg.2750	hypothetical protein	fig|2045.18.peg.677	99.49
189	fig|2045.14.peg.2766	integrase family protein	fig|2045.18.peg.694	99.46
151	fig|2045.14.peg.2871	hypothetical protein	fig|2045.18.peg.793	99.33
412	fig|2045.14.peg.2993	hypothetical protein	fig|2045.18.peg.915	99.68
467	fig|2045.14.peg.3001	hypothetical protein	fig|2045.18.peg.923	99.79
301	fig|2045.14.peg.3002	hypothetical protein	fig|2045.18.peg.924	99.67
351	fig|2045.14.peg.3004	ABC transporter, permease protein (cluster 8, B12/iron complex)	fig|2045.18.peg.925	99.71
538	fig|2045.14.peg.3286	P-loop GTPase domain-containing protein Sros_8871	fig|2045.18.peg.1208	97.17
463	fig|2045.14.peg.3297	hypothetical protein	fig|2045.18.peg.1219	99.7
160	fig|2045.14.peg.3420	hypothetical protein	fig|2045.18.peg.1343	99.37
59	fig|2045.14.peg.3516	hypothetical protein	fig|2045.18.peg.1439	97.92
400	fig|2045.14.peg.3590	Cell division protein FtsZ	fig|2045.18.peg.1513	99.25
865	fig|2045.14.peg.3744	hypothetical protein	fig|2045.18.peg.1667	99.88
167	fig|2045.14.peg.4017	hypothetical protein	fig|2045.18.peg.1938	99.4
868	fig|2045.14.peg.4107	Acetoacetyl-CoA synthetase (EC 6.2.1.16)	fig|2045.18.peg.2028	99.88
452	fig|2045.14.peg.4282	16S rRNA (cytosine(967)-C(5))-methyltransferase (EC 2.1.1.176)	fig|2045.18.peg.2203	98.9
1137	fig|2045.14.peg.4416	hypothetical protein	fig|2045.18.peg.2338	98.42
201	fig|2045.14.peg.4489	Phage tail fiber protein	fig|2045.18.peg.2412	99.5
70	fig|2045.14.peg.4553	hypothetical protein	fig|2045.18.peg.2477	98.55
818	fig|2045.14.peg.4589	DNA translocase FtsK	fig|2045.18.peg.2513	99.87

43	fig|2045.14.peg.4617	hypothetical protein	fig|2045.18.peg.27	89.29
56	fig|2045.14.peg.4625	hypothetical protein	fig|2045.18.peg.2550	98.18
1984	fig|2045.14.peg.4659	Relaxase TrwC / Conjugal transfer protein TraA / DNA primase (EC 2.7.7.-), conjugative	fig|2045.18.peg.2584	99.09
95	fig|2045.14.peg.4764	ATP/GTP-binding protein	fig|2045.18.peg.2688	98.94
235	fig|2045.14.peg.4799	Integral membrane protein	fig|2045.18.peg.2723	99.57
1063	fig|2045.14.peg.5043	Putative large secreted protein SCO0341	fig|2045.18.peg.2968	99.9
476	fig|2045.14.peg.5150	Molybdopterin-binding oxidoreductase, [4Fe-4S] cluster binding and molybdopterin cofactor binding site containing	fig|2045.18.peg.3075	99.79
409	fig|2045.14.peg.5360	FIG01111984: hypothetical protein	fig|2045.18.peg.3285	95.83
293	fig|2045.14.peg.5432	hypothetical protein	fig|2045.18.peg.3357	99.66
283	fig|2045.14.peg.5463	hypothetical protein	fig|2045.18.peg.3388	99.29
185	fig|2045.14.peg.5464	hypothetical protein	fig|2045.18.peg.3389	98.48
114	fig|2045.14.peg.5510	hypothetical protein	fig|2045.18.peg.3437	98.63
126	fig|2045.14.peg.5625	FIG01121360: hypothetical protein	fig|2045.18.peg.3553	98.97
955	fig|2045.14.peg.5629	Glycosyl transferase, family 2	fig|2045.18.peg.3557	99.9
294	fig|2045.14.peg.5650	Transcriptional regulator, LysR family	fig|2045.18.peg.3578	99.66
407	fig|2045.14.peg.5652	Cell envelope-associated transcriptional attenuator LytR-CpsA-Psr, subfamily A1	fig|2045.18.peg.3580	99.67
306	fig|2045.14.peg.506	Beta-glucanase precursor (EC 3.2.1.73)	fig|2045.18.peg.343	56.03
289	fig|2045.14.peg.709	hypothetical protein	fig|2045.18.peg.4636	99.65

Table S2. The genomic SNP/indel analysis between strain N39 and N40

Location of SNP/indel site in N39	The base in N39	The base in N40	Location of SNP/indel site in N40	Contig of SNP/indel site in N39	Contig of SNP/indel site in N40
61194	G	T	3824609	N39-chromosome	N40-chromosome
72818	A	T	3836233	N39-chromosome	N40-chromosome
72821	A	C	3836236	N39-chromosome	N40-chromosome
101455	G	.	3864869	N39-chromosome	N40-chromosome
101457	G	C	3864871	N39-chromosome	N40-chromosome
101458	G	T	3864872	N39-chromosome	N40-chromosome
101464	C	G	3864878	N39-chromosome	N40-chromosome
101466	C	A	3864880	N39-chromosome	N40-chromosome
101470	G	C	3864884	N39-chromosome	N40-chromosome
101474	G	.	3864887	N39-chromosome	N40-chromosome
101475	C	.	3864887	N39-chromosome	N40-chromosome
101476	C	.	3864887	N39-chromosome	N40-chromosome
101479	C	A	3864890	N39-chromosome	N40-chromosome
343931	C	.	4107341	N39-chromosome	N40-chromosome
343932	C	T	4107342	N39-chromosome	N40-chromosome
407251	G	.	4170660	N39-chromosome	N40-chromosome
466189	G	.	4229597	N39-chromosome	N40-chromosome
689383	G	.	4452790	N39-chromosome	N40-chromosome
748087	.	G	4511495	N39-chromosome	N40-chromosome
748281	.	G	4511690	N39-chromosome	N40-chromosome
751397	.	C	4514807	N39-chromosome	N40-chromosome
751397	.	G	4514808	N39-chromosome	N40-chromosome
876999	G	A	4640410	N39-chromosome	N40-chromosome
985655	C	G	4749066	N39-chromosome	N40-chromosome
1290993	A	C	5054404	N39-chromosome	N40-chromosome
1449191	G	.	5212629	N39-chromosome	N40-chromosome
1919982	A	G	5683420	N39-chromosome	N40-chromosome
2189763	.	T	5951759	N39-chromosome	N40-chromosome
2264647	.	G	6026644	N39-chromosome	N40-chromosome
2264647	.	C	6026645	N39-chromosome	N40-chromosome
2264647	.	C	6026646	N39-chromosome	N40-chromosome
2264647	.	C	6026647	N39-chromosome	N40-chromosome
2264647	.	C	6026648	N39-chromosome	N40-chromosome
2264647	.	C	6026649	N39-chromosome	N40-chromosome
2264647	.	C	6026650	N39-chromosome	N40-chromosome
2264647	.	C	6026651	N39-chromosome	N40-chromosome
2264647	.	C	6026652	N39-chromosome	N40-chromosome
2264647	.	C	6026653	N39-chromosome	N40-chromosome

2264647	.	C	6026654	N39-chromosome	N40-chromosome
2264647	.	C	6026655	N39-chromosome	N40-chromosome
2264647	.	C	6026656	N39-chromosome	N40-chromosome
2264647	.	C	6026657	N39-chromosome	N40-chromosome
2264647	.	C	6026658	N39-chromosome	N40-chromosome
2264647	.	C	6026659	N39-chromosome	N40-chromosome
2264647	.	G	6026660	N39-chromosome	N40-chromosome
2264647	.	G	6026661	N39-chromosome	N40-chromosome
2264655	T	G	6026669	N39-chromosome	N40-chromosome
2264656	C	G	6026670	N39-chromosome	N40-chromosome
2264658	T	G	6026672	N39-chromosome	N40-chromosome
2264659	.	G	6026674	N39-chromosome	N40-chromosome
2264659	.	G	6026675	N39-chromosome	N40-chromosome
2264659	.	G	6026676	N39-chromosome	N40-chromosome
2264659	.	G	6026677	N39-chromosome	N40-chromosome
2264659	.	G	6026678	N39-chromosome	N40-chromosome
2264659	.	C	6026679	N39-chromosome	N40-chromosome
2264659	.	C	6026680	N39-chromosome	N40-chromosome
2264659	.	C	6026681	N39-chromosome	N40-chromosome
2264659	.	C	6026682	N39-chromosome	N40-chromosome
2264659	.	C	6026683	N39-chromosome	N40-chromosome
2264661	G	C	6026685	N39-chromosome	N40-chromosome
2264662	A	C	6026686	N39-chromosome	N40-chromosome
2414870	C	.	85899	N39-chromosome	N40-chromosome
2414871	A	.	85899	N39-chromosome	N40-chromosome
2778514	G	.	449541	N39-chromosome	N40-chromosome
3078737	.	C	748330	N39-chromosome	N40-chromosome
3078737	.	C	748331	N39-chromosome	N40-chromosome
3090687	.	G	760282	N39-chromosome	N40-chromosome
3091745	G	.	761339	N39-chromosome	N40-chromosome
3091746	A	.	761339	N39-chromosome	N40-chromosome
3091762	C	G	761355	N39-chromosome	N40-chromosome
3091834	.	C	761428	N39-chromosome	N40-chromosome
3227568	C	.	897161	N39-chromosome	N40-chromosome
3399408	C	.	1069000	N39-chromosome	N40-chromosome
3399409	G	.	1069000	N39-chromosome	N40-chromosome
3411751	G	.	1081341	N39-chromosome	N40-chromosome
3437540	G	T	1107130	N39-chromosome	N40-chromosome
3459593	G	.	1129182	N39-chromosome	N40-chromosome
3561499	G	.	1231087	N39-chromosome	N40-chromosome
3697655	A	.	1367242	N39-chromosome	N40-chromosome
3697660	.	G	1367248	N39-chromosome	N40-chromosome
4014681	G	A	1684269	N39-chromosome	N40-chromosome

4105367	A	C	1773829	N39-chromosome	N40-chromosome
4393721	G	C	2062183	N39-chromosome	N40-chromosome
4393723	.	C	2062186	N39-chromosome	N40-chromosome
4393723	.	C	2062187	N39-chromosome	N40-chromosome
4393723	.	C	2062188	N39-chromosome	N40-chromosome
4393723	.	C	2062189	N39-chromosome	N40-chromosome
4393723	.	C	2062190	N39-chromosome	N40-chromosome
4393723	.	C	2062191	N39-chromosome	N40-chromosome
4393723	.	C	2062192	N39-chromosome	N40-chromosome
4393723	.	C	2062193	N39-chromosome	N40-chromosome
4393723	.	C	2062194	N39-chromosome	N40-chromosome
4393723	.	C	2062195	N39-chromosome	N40-chromosome
4393723	.	C	2062196	N39-chromosome	N40-chromosome
4393723	.	C	2062197	N39-chromosome	N40-chromosome
4393723	.	C	2062198	N39-chromosome	N40-chromosome
4393723	.	T	2062199	N39-chromosome	N40-chromosome
4393726	G	T	2062202	N39-chromosome	N40-chromosome
4393728	C	A	2062204	N39-chromosome	N40-chromosome
4393729	G	A	2062205	N39-chromosome	N40-chromosome
4393732	G	A	2062208	N39-chromosome	N40-chromosome
4393733	.	A	2062210	N39-chromosome	N40-chromosome
4393733	.	A	2062211	N39-chromosome	N40-chromosome
4393737	C	G	2062215	N39-chromosome	N40-chromosome
4393738	.	C	2062217	N39-chromosome	N40-chromosome
4393738	.	A	2062218	N39-chromosome	N40-chromosome
4393738	.	T	2062219	N39-chromosome	N40-chromosome
4393738	.	A	2062220	N39-chromosome	N40-chromosome
4393738	.	C	2062221	N39-chromosome	N40-chromosome
4393740	.	G	2062224	N39-chromosome	N40-chromosome
4393740	.	A	2062225	N39-chromosome	N40-chromosome
4393740	.	T	2062226	N39-chromosome	N40-chromosome
4393740	.	T	2062227	N39-chromosome	N40-chromosome
4393740	.	A	2062228	N39-chromosome	N40-chromosome
4393740	.	G	2062229	N39-chromosome	N40-chromosome
4393746	.	T	2062236	N39-chromosome	N40-chromosome
4393747	.	A	2062238	N39-chromosome	N40-chromosome
4393747	.	C	2062239	N39-chromosome	N40-chromosome
4393749	C	G	2062241	N39-chromosome	N40-chromosome
4393752	.	T	2062245	N39-chromosome	N40-chromosome
4393753	C	T	2062246	N39-chromosome	N40-chromosome
4393757	.	C	2062251	N39-chromosome	N40-chromosome
4393757	.	G	2062252	N39-chromosome	N40-chromosome
4393757	.	T	2062253	N39-chromosome	N40-chromosome

4393757	.	G	2062254	N39-chromosome	N40-chromosome
4393757	.	T	2062255	N39-chromosome	N40-chromosome
4393760	.	C	2062259	N39-chromosome	N40-chromosome
4393760	.	T	2062260	N39-chromosome	N40-chromosome
4393760	.	T	2062261	N39-chromosome	N40-chromosome
4393760	.	C	2062262	N39-chromosome	N40-chromosome
4393762	.	A	2062265	N39-chromosome	N40-chromosome
4393762	.	T	2062266	N39-chromosome	N40-chromosome
4393762	.	C	2062267	N39-chromosome	N40-chromosome
4393763	.	G	2062269	N39-chromosome	N40-chromosome
4393763	.	G	2062270	N39-chromosome	N40-chromosome
4393763	.	C	2062271	N39-chromosome	N40-chromosome
4393775	A	C	2062283	N39-chromosome	N40-chromosome
4705674	.	C	2375559	N39-chromosome	N40-chromosome
4707326	.	G	2377212	N39-chromosome	N40-chromosome
4734300	G	C	2404186	N39-chromosome	N40-chromosome
4734312	C	G	2404198	N39-chromosome	N40-chromosome
4734317	A	G	2404203	N39-chromosome	N40-chromosome
4734351	C	G	2404237	N39-chromosome	N40-chromosome
4734384	C	A	2404270	N39-chromosome	N40-chromosome
4734393	C	G	2404279	N39-chromosome	N40-chromosome
4734426	C	G	2404312	N39-chromosome	N40-chromosome
4734438	C	T	2404324	N39-chromosome	N40-chromosome
4734462	C	G	2404348	N39-chromosome	N40-chromosome
4734477	C	G	2404363	N39-chromosome	N40-chromosome
4734594	T	G	2404480	N39-chromosome	N40-chromosome
4734699	C	.	2404584	N39-chromosome	N40-chromosome
4734701	.	T	2404587	N39-chromosome	N40-chromosome
4734866	A	G	2404752	N39-chromosome	N40-chromosome
4734897	G	T	2404783	N39-chromosome	N40-chromosome
4734987	A	C	2404873	N39-chromosome	N40-chromosome
4735014	C	A	2404900	N39-chromosome	N40-chromosome
4735015	C	G	2404901	N39-chromosome	N40-chromosome
4735017	C	T	2404903	N39-chromosome	N40-chromosome
4735034	A	T	2404920	N39-chromosome	N40-chromosome
4735056	A	C	2404942	N39-chromosome	N40-chromosome
4735069	A	C	2404955	N39-chromosome	N40-chromosome
4735074	C	A	2404960	N39-chromosome	N40-chromosome
4735497	G	T	2405383	N39-chromosome	N40-chromosome
4735503	C	T	2405389	N39-chromosome	N40-chromosome
4735713	C	G	2405599	N39-chromosome	N40-chromosome
4735836	C	A	2405722	N39-chromosome	N40-chromosome
4735956	C	T	2405842	N39-chromosome	N40-chromosome

4735962	C	T	2405848	N39-chromosome	N40-chromosome
4736169	A	G	2406055	N39-chromosome	N40-chromosome
4736193	C	T	2406079	N39-chromosome	N40-chromosome
4736220	A	G	2406106	N39-chromosome	N40-chromosome
4736254	C	A	2406140	N39-chromosome	N40-chromosome
4736343	C	G	2406229	N39-chromosome	N40-chromosome
4736406	C	G	2406292	N39-chromosome	N40-chromosome
4736429	C	G	2406315	N39-chromosome	N40-chromosome
4736478	C	G	2406364	N39-chromosome	N40-chromosome
4736499	C	G	2406385	N39-chromosome	N40-chromosome
4736556	C	G	2406442	N39-chromosome	N40-chromosome
4736559	C	G	2406445	N39-chromosome	N40-chromosome
4736586	C	G	2406472	N39-chromosome	N40-chromosome
4736589	C	A	2406475	N39-chromosome	N40-chromosome
4736610	A	G	2406496	N39-chromosome	N40-chromosome
4736619	C	G	2406505	N39-chromosome	N40-chromosome
4736646	C	T	2406532	N39-chromosome	N40-chromosome
4736650	G	C	2406536	N39-chromosome	N40-chromosome
4736661	C	T	2406547	N39-chromosome	N40-chromosome
4736718	G	C	2406604	N39-chromosome	N40-chromosome
4736721	C	G	2406607	N39-chromosome	N40-chromosome
4736725	A	G	2406611	N39-chromosome	N40-chromosome
4736729	C	G	2406615	N39-chromosome	N40-chromosome
4736730	G	C	2406616	N39-chromosome	N40-chromosome
4736739	A	C	2406625	N39-chromosome	N40-chromosome
4736742	G	C	2406628	N39-chromosome	N40-chromosome
4736743	G	C	2406629	N39-chromosome	N40-chromosome
4736746	C	G	2406632	N39-chromosome	N40-chromosome
4736763	G	C	2406649	N39-chromosome	N40-chromosome
4736766	C	G	2406652	N39-chromosome	N40-chromosome
4736771	G	A	2406657	N39-chromosome	N40-chromosome
4736773	A	G	2406659	N39-chromosome	N40-chromosome
4736775	C	G	2406661	N39-chromosome	N40-chromosome
4736793	G	C	2406679	N39-chromosome	N40-chromosome
4736796	A	C	2406682	N39-chromosome	N40-chromosome
4736811	G	C	2406697	N39-chromosome	N40-chromosome
4736814	G	T	2406700	N39-chromosome	N40-chromosome
4736842	T	C	2406728	N39-chromosome	N40-chromosome
4736859	A	C	2406745	N39-chromosome	N40-chromosome
4736868	C	G	2406754	N39-chromosome	N40-chromosome
4736886	A	C	2406772	N39-chromosome	N40-chromosome
4736889	A	G	2406775	N39-chromosome	N40-chromosome
4736893	G	A	2406779	N39-chromosome	N40-chromosome

4736901	C	G	2406787	N39-chromosome	N40-chromosome
4736916	G	A	2406802	N39-chromosome	N40-chromosome
4736958	C	G	2406844	N39-chromosome	N40-chromosome
4736991	C	G	2406877	N39-chromosome	N40-chromosome
4737003	G	A	2406889	N39-chromosome	N40-chromosome
4737010	C	G	2406896	N39-chromosome	N40-chromosome
4737021	C	G	2406907	N39-chromosome	N40-chromosome
4737039	C	T	2406925	N39-chromosome	N40-chromosome
4737049	G	T	2406935	N39-chromosome	N40-chromosome
4962724	C	A	2632610	N39-chromosome	N40-chromosome
4962730	T	G	2632616	N39-chromosome	N40-chromosome
5036806	G	.	2706691	N39-chromosome	N40-chromosome
5036807	A	.	2706691	N39-chromosome	N40-chromosome
5134660	G	.	2804543	N39-chromosome	N40-chromosome
5134661	C	.	2804543	N39-chromosome	N40-chromosome
5134662	C	.	2804543	N39-chromosome	N40-chromosome
5134663	G	.	2804543	N39-chromosome	N40-chromosome
5247818	G	C	2917698	N39-chromosome	N40-chromosome
5349832	G	C	3019712	N39-chromosome	N40-chromosome
5457830	G	.	3127709	N39-chromosome	N40-chromosome
5457831	G	.	3127709	N39-chromosome	N40-chromosome
5457832	C	.	3127709	N39-chromosome	N40-chromosome
5457833	G	.	3127709	N39-chromosome	N40-chromosome
5457834	C	.	3127709	N39-chromosome	N40-chromosome
5457835	G	.	3127709	N39-chromosome	N40-chromosome
5457836	G	.	3127709	N39-chromosome	N40-chromosome
5457837	G	.	3127709	N39-chromosome	N40-chromosome
5457841	G	.	3127712	N39-chromosome	N40-chromosome
5457842	G	.	3127712	N39-chromosome	N40-chromosome
5457843	C	.	3127712	N39-chromosome	N40-chromosome
5457844	C	.	3127712	N39-chromosome	N40-chromosome
5457845	G	.	3127712	N39-chromosome	N40-chromosome
5457846	G	.	3127712	N39-chromosome	N40-chromosome
5457847	A	.	3127712	N39-chromosome	N40-chromosome
5457848	G	.	3127712	N39-chromosome	N40-chromosome
5457849	C	.	3127712	N39-chromosome	N40-chromosome
5457850	G	.	3127712	N39-chromosome	N40-chromosome
5457851	G	.	3127712	N39-chromosome	N40-chromosome
5457852	C	.	3127712	N39-chromosome	N40-chromosome
5457853	C	.	3127712	N39-chromosome	N40-chromosome
5457854	G	.	3127712	N39-chromosome	N40-chromosome
5457855	G	.	3127712	N39-chromosome	N40-chromosome
5457856	G	.	3127712	N39-chromosome	N40-chromosome

5457860	T	.	3127715	N39-chromosome	N40-chromosome
5457861	G	.	3127715	N39-chromosome	N40-chromosome
5457862	C	.	3127715	N39-chromosome	N40-chromosome
5457863	T	.	3127715	N39-chromosome	N40-chromosome
5457864	G	.	3127715	N39-chromosome	N40-chromosome
5457865	C	.	3127715	N39-chromosome	N40-chromosome
5457866	T	.	3127715	N39-chromosome	N40-chromosome
5457867	G	.	3127715	N39-chromosome	N40-chromosome
5457868	C	.	3127715	N39-chromosome	N40-chromosome
5457869	G	.	3127715	N39-chromosome	N40-chromosome
5457870	G	.	3127715	N39-chromosome	N40-chromosome
5457871	G	.	3127715	N39-chromosome	N40-chromosome
5457872	A	.	3127715	N39-chromosome	N40-chromosome
5457873	C	.	3127715	N39-chromosome	N40-chromosome
5457874	G	.	3127715	N39-chromosome	N40-chromosome
5457875	G	.	3127715	N39-chromosome	N40-chromosome
5457876	G	.	3127715	N39-chromosome	N40-chromosome
5457878	T	.	3127716	N39-chromosome	N40-chromosome
5563526	C	G	3233364	N39-chromosome	N40-chromosome
5563528	T	C	3233366	N39-chromosome	N40-chromosome
5563529	G	T	3233367	N39-chromosome	N40-chromosome
5563531	T	G	3233369	N39-chromosome	N40-chromosome
5564464	C	.	3234301	N39-chromosome	N40-chromosome
5564522	C	.	3234358	N39-chromosome	N40-chromosome
5564523	C	.	3234358	N39-chromosome	N40-chromosome
5564524	C	.	3234358	N39-chromosome	N40-chromosome
5564525	C	.	3234358	N39-chromosome	N40-chromosome
5741184	C	A	3412104	N39-chromosome	N40-chromosome
5764887	T	G	3435807	N39-chromosome	N40-chromosome
5765632	C	.	3436551	N39-chromosome	N40-chromosome
5843024	.	G	3513944	N39-chromosome	N40-chromosome
5843024	.	G	3513945	N39-chromosome	N40-chromosome
5843024	.	C	3513946	N39-chromosome	N40-chromosome
5843024	.	G	3513947	N39-chromosome	N40-chromosome
5843024	.	C	3513948	N39-chromosome	N40-chromosome
5843024	.	G	3513949	N39-chromosome	N40-chromosome
5843024	.	G	3513950	N39-chromosome	N40-chromosome
5843024	.	G	3513951	N39-chromosome	N40-chromosome
5843024	.	C	3513952	N39-chromosome	N40-chromosome
5843024	.	C	3513953	N39-chromosome	N40-chromosome
5843024	.	C	3513954	N39-chromosome	N40-chromosome
5843024	.	C	3513955	N39-chromosome	N40-chromosome
5843024	.	C	3513956	N39-chromosome	N40-chromosome

5843024	.	C	3513957	N39-chromosome	N40-chromosome
5843024	.	C	3513958	N39-chromosome	N40-chromosome
5843024	.	C	3513959	N39-chromosome	N40-chromosome
5843024	.	C	3513960	N39-chromosome	N40-chromosome
5843024	.	C	3513961	N39-chromosome	N40-chromosome
5843024	.	C	3513962	N39-chromosome	N40-chromosome
5843024	.	C	3513963	N39-chromosome	N40-chromosome
5843024	.	C	3513964	N39-chromosome	N40-chromosome
5843024	.	C	3513965	N39-chromosome	N40-chromosome
5843024	.	C	3513966	N39-chromosome	N40-chromosome
5843024	.	C	3513967	N39-chromosome	N40-chromosome
5843024	.	G	3513968	N39-chromosome	N40-chromosome
5843024	.	G	3513969	N39-chromosome	N40-chromosome
5843024	.	G	3513970	N39-chromosome	N40-chromosome
5843024	.	G	3513971	N39-chromosome	N40-chromosome
5843024	.	G	3513972	N39-chromosome	N40-chromosome
5843024	.	G	3513973	N39-chromosome	N40-chromosome
5843024	.	G	3513974	N39-chromosome	N40-chromosome
5843024	.	G	3513975	N39-chromosome	N40-chromosome
5843024	.	G	3513976	N39-chromosome	N40-chromosome
5843024	.	C	3513977	N39-chromosome	N40-chromosome
5843024	.	G	3513978	N39-chromosome	N40-chromosome
5843024	.	C	3513979	N39-chromosome	N40-chromosome
5843024	.	G	3513980	N39-chromosome	N40-chromosome
5843024	.	G	3513981	N39-chromosome	N40-chromosome
5843024	.	C	3513982	N39-chromosome	N40-chromosome
5843024	.	C	3513983	N39-chromosome	N40-chromosome
5843024	.	C	3513984	N39-chromosome	N40-chromosome
5843024	.	C	3513985	N39-chromosome	N40-chromosome
5843024	.	G	3513986	N39-chromosome	N40-chromosome
5843024	.	A	3513987	N39-chromosome	N40-chromosome
5843024	.	C	3513988	N39-chromosome	N40-chromosome
5843024	.	T	3513989	N39-chromosome	N40-chromosome
5843024	.	C	3513990	N39-chromosome	N40-chromosome
5843024	.	G	3513991	N39-chromosome	N40-chromosome
5843024	.	A	3513992	N39-chromosome	N40-chromosome
5843024	.	G	3513993	N39-chromosome	N40-chromosome
5843024	.	C	3513994	N39-chromosome	N40-chromosome
5843024	.	G	3513995	N39-chromosome	N40-chromosome
5843024	.	A	3513996	N39-chromosome	N40-chromosome
5843024	.	G	3513997	N39-chromosome	N40-chromosome
5843024	.	T	3513998	N39-chromosome	N40-chromosome
5843024	.	C	3513999	N39-chromosome	N40-chromosome

5843024	.	G	3514000	N39-chromosome	N40-chromosome
5843024	.	G	3514001	N39-chromosome	N40-chromosome
5843024	.	G	3514002	N39-chromosome	N40-chromosome
5843024	.	G	3514003	N39-chromosome	N40-chromosome
5843024	.	G	3514004	N39-chromosome	N40-chromosome
5843024	.	C	3514005	N39-chromosome	N40-chromosome
5843024	.	C	3514006	N39-chromosome	N40-chromosome
5843024	.	G	3514007	N39-chromosome	N40-chromosome
5843024	.	C	3514008	N39-chromosome	N40-chromosome
5843024	.	G	3514009	N39-chromosome	N40-chromosome
5843024	.	G	3514010	N39-chromosome	N40-chromosome
5843024	.	C	3514011	N39-chromosome	N40-chromosome
5843024	.	G	3514012	N39-chromosome	N40-chromosome
5843026	.	G	3514015	N39-chromosome	N40-chromosome
5843026	.	T	3514016	N39-chromosome	N40-chromosome
5843026	.	A	3514017	N39-chromosome	N40-chromosome

Note: the point in the table represents a base deletion.

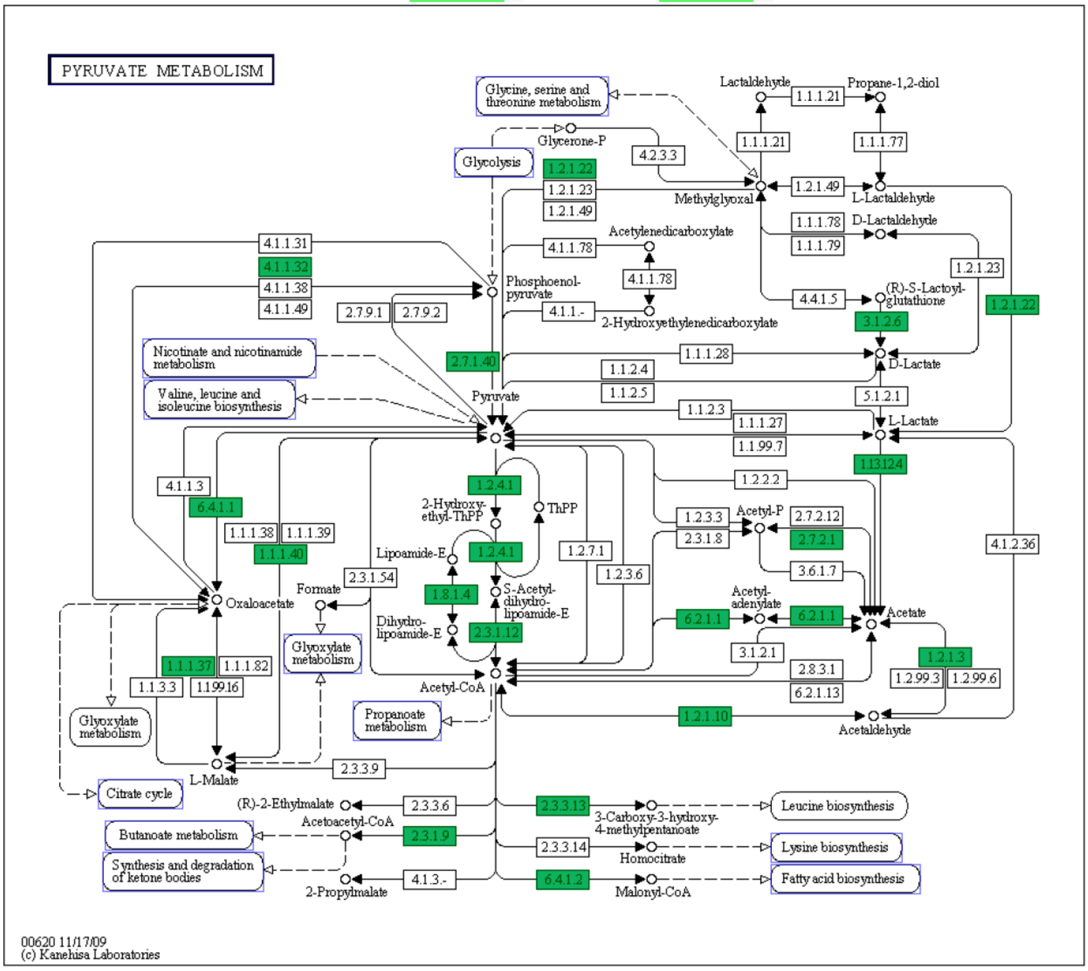

Figure S1 The metabolic pathway of pyruvate in N39 obtained from KEGG database.

Green box represents the enzyme annotated in genome of N39.

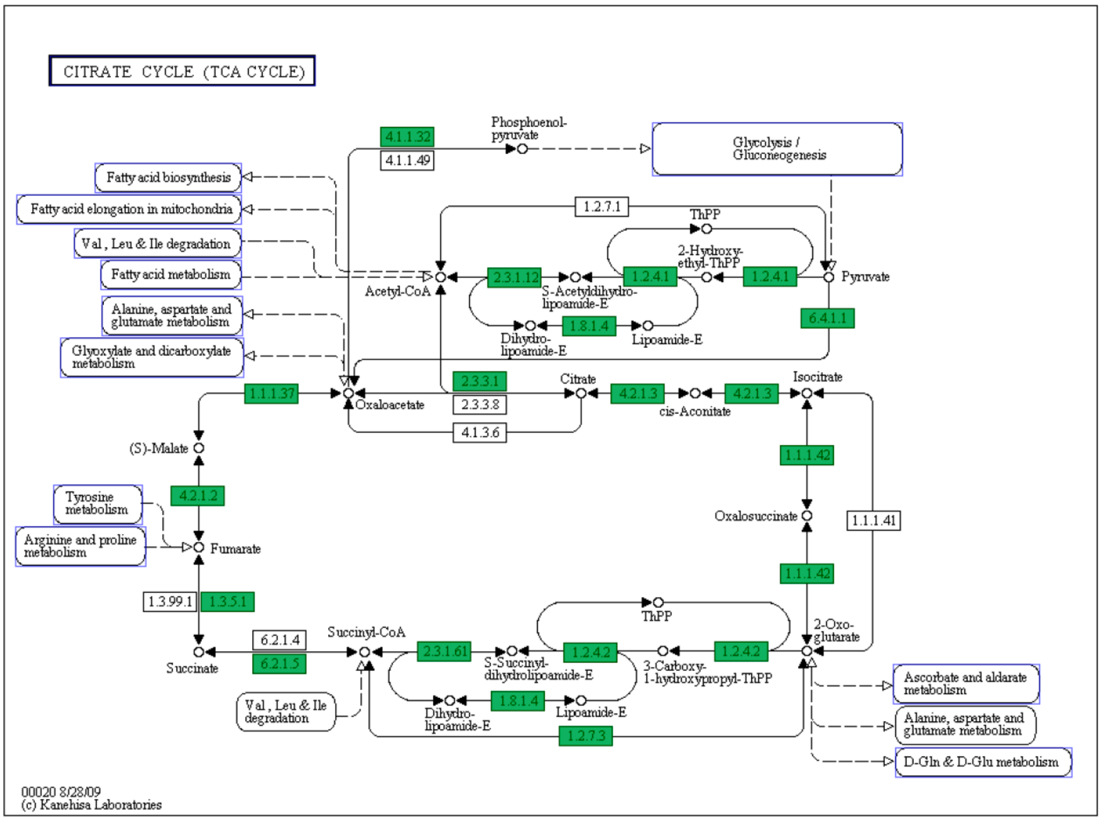

Figure S2 The metabolic pathway of citrate cycle in N39 obtained from KEGG database. Green box represents the enzyme annotated in genome of N39.
